# Skeletal muscle deficits are associated with worse exercise performance in pediatric pulmonary hypertension

**DOI:** 10.3389/fped.2022.1025420

**Published:** 2022-10-05

**Authors:** Catherine M. Avitabile, Michael G. McBride, Matthew A. Harris, Kevin K. Whitehead, Mark A. Fogel, Stephen M. Paridon, Babette S. Zemel

**Affiliations:** ^1^Division of Cardiology, Children’s Hospital of Philadelphia, Philadelphia, PA, United States; ^2^Department of Pediatrics, University of Pennsylvania Perelman School of Medicine, Philadelphia, PA, United States; ^3^Division of Gastroenterology, Hepatology, and Nutrition, Children’s Hospital of Philadelphia, Philadelphia, PA, United States

**Keywords:** pediatric pulmonary hypertension, skeletal muscle, exercise, densitometry, cardiac magnetic resonance imaging

## Abstract

**Background:**

Skeletal muscle deficits are associated with worse exercise performance in adults with pulmonary hypertension (PH) but the impact is poorly understood in pediatric PH.

**Objective:**

To study muscle deficits, physical inactivity, and performance on cardiopulmonary exercise test (CPET) and exercise cardiac magnetic resonance (eCMR) in pediatric PH.

**Methods:**

Youth 8–18 years participated in a prospective, cross-sectional study including densitometry (DXA) for measurement of leg lean mass Z-score (LLMZ), handheld dynamometer with generation of dominant and non-dominant handgrip Z-scores, Physical Activity Questionnaire (PAQ), CPET, and optional eCMR. CPET parameters were expressed relative to published reference values. CMR protocol included ventricular volumes and indexed systemic flow at rest and just after supine ergometer exercise. Relationships between LLMZ, PAQ score, and exercise performance were assessed by Pearson correlation and multiple linear regression.

**Results:**

There were 25 participants (13.7 ± 2.8 years, 56% female, 64% PH Group 1, 60% functional class I); 12 (48%) performed both CPET and eCMR. Mean LLMZ (–0.96 ± 1.14) was associated with PAQ score (*r* = 50, *p* = 0.01) and with peak oxygen consumption (VO_2_) (*r* = 0.74, *p* = < 0.001), VO_2_ at anaerobic threshold (*r* = 0.65, *p* < 0.001), and peak work rate (*r* = 0.64, *p* < 0.01). Higher handgrip Z-scores were associated with better CPET and eCMR performance. On regression analysis, LLMZ and PAQ score were positively associated with peak VO_2_, while handgrip Z-score and PAQ score were positively associated with peak work rate.

**Conclusion:**

Muscle mass and strength are positively associated with exercise performance in pediatric PH. Future studies should determine the effect of rehabilitation programs on muscle properties and exercise performance.

## Introduction

Pediatric pulmonary hypertension (PH) is associated with various vascular, cardiac, pulmonary, and systemic conditions. Without treatment, the disease leads to right ventricular dysfunction, right ventricular failure, and death. While therapies have improved in recent years (1, 2), long-term outcomes remain poor. Patients report low quality of life in the face of significant morbidity and mortality risk (3, 4). Exercise intolerance is common in PH patients, significantly impacting quality of life and prognosis. While cardiopulmonary status affects exercise performance, the contribution of peripheral factors, including skeletal muscle dysfunction, is increasingly recognized (5). Skeletal muscle dysfunction is associated with worse 6-min walk distance (6 MWD) in adults with idiopathic pulmonary arterial hypertension (IPAH) (6). Similarly, we previously reported marked deficits in densitometry (DXA)-measured leg lean mass, a surrogate marker of skeletal muscle, in youth with PH in association with inactivity and worse 6 MWD (7). In other studies, skeletal muscle atrophy, impaired peripheral oxygen extraction, and reduced muscle contractility suggest that PH patients exhibit a generalized “myopathy” similar to patients with left heart failure (8–11). The skeletal muscle pump is critically important to augmentation of systemic venous return and pulmonary blood flow with erect exercise (12). Skeletal muscle dysfunction may critically hinder this mechanism in PH patients. Therefore, we sought to expand on our prior findings by characterizing leg lean mass Z-score (LLMZ) and muscle strength in a different cohort of pediatric PH patients and to explore the associations between muscle deficits, self-reported physical activity, and measures of exercise performance on cardiopulmonary exercise test (CPET) and exercise cardiac magnetic resonance (eCMR).

## Materials and methods

### Study population

Youth ages 8–18 years with World Symposium of PH (WSPH) diagnostic Groups 1–4 and functional class I or II were prospectively enrolled in a cross-sectional study from 2018 to 2021. Exclusion criteria included pregnancy, functional class III or IV, single ventricle physiology, moderate to severe chronic kidney disease (stage 3 or greater), severe hepatic impairment (transaminases > 2 times the upper limit of normal), pacemaker, defibrillator, or other metal that would interfer with imaging, and significant developmental delay or inability to comply with verbal English instructions in order to complete the study procedures. Fully informed, written consent was obtained from the parent/legal guardian of participants < 18 years and of participants 18 years of age. In addition, age-appropriate informed assent was obtained from participants < 18 years. This study was approved by the Children’s Hospital of Philadelphia Institutional Review Board.

### Study procedures

#### Pulmonary hypertension history and standard of care testing

The medical record was queried for WSPH diagnostic group, World Health Organization (WHO) functional class, medications, and standard of care testing including last echocardiogram, 6-min walk test (within 3 months), cardiac catheterization data, and brain-type natriuretic peptide. Six-minute walk tests were performed according to American Thoracic Society guidelines (13).

#### Anthropometry and tanner stage

Weight was measured to the nearest 0.1 kg with a digital electronic stand-on scale. Height and sitting height were measured to the nearest 0.1 cm with a wall-mounted stadiometer in order to calculate leg length (leg length = height - sitting height). Tanner stage was determined via a validated self-assessment tool (14).

#### Heath-related quality of life

Participants and parents/guardians completed the Pediatric Cardiac Quality of Life Inventory, a reliable and validated instrument of disease specific quality of life for patients 8–18 years of age with congenital or acquired heart disease (15). Disease impact and psychosocial impact subscores were summed to generate a total score with higher scores (maximum 100 points) representing better health-related quality of life.

#### Physical activity questionnaire

Participants completed the Physical Activity Questionnaire (PAQ) for Older Children or Adolescents (PAQ-C and PAQ-A), 7-day recall instruments designed to assess moderate to vigorous physical activity (16–18). The 5-point scoring scale was used to calculate a final summary score from the means of scores for each question. Average scores > 3 are reported in healthy populations (16).

#### Vitamin D levels

Quantification of circulating 25 (OH) vitamin D was performed by HPLC tandem mass-spectrometry (19). Vitamin D deficiency was defined as serum level less than 20 ng/mL (20).

#### Body composition

Whole body lean and fat mass were measured with a Hologic Delphi densitometer (Bedford, Massachusetts, USA) in array mode (software V.12.4). Measurements were performed with standard supine positioning techniques with participants wearing scrubs to minimize scan variability. Urine pregnancy test was performed prior to DXA in female participants. Whole body lean mass was calculated as fat-free mass minus bone mineral content. Leg lean mass was used as a measure of skeletal muscle, given the previously reported concerns with the use of whole body lean mass as a representation of muscle mass (21). Calibration was performed daily with a hydroxyapatite phantom and weekly with a whole-body phantom. Coefficients of variation ranged from 1 to 4% (22).

#### Muscle strength testing

Bilateral forearm strength was measured with a handgrip dynamometer (Takei, Tokyo, Japan) (23). Hand dominance was determined by which hand the participant used to hold a pencil. The participant stood upright with the shoulder adducted holding the dynamometer, not touching the trunk. The handle was adjusted to the hand size of the participant, and no extra body movement was allowed during testing. For each hand, 3 maximal effort trials lasting 4–5 s interspersed with 60-s rests were carried out. The highest value was retained for analysis. Lower extremity strength (knee and ankle) was assessed using the Biodex Multi-Joint System 3 Pro (Biodex Medical Systems, Inc., Shirley, NY, USA) (24). For the knee, peak quadricep muscle torque (ft-lbs) was measured in knee flexion and extension. Participants sat with their thighs at an angle of 110° to the trunk. The trunk and both thighs were stabilized with belts. The tested knee was positioned at 90° flexion, and the mechanical axis of the dynamometer was aligned with the lateral epicondyle of the knee. Each participant performed 10 concentric contractions at 120°/s (flexion and extension) of both sides, and the highest value was recorded. For the ankle, peak calf muscle torque (ft-lbs) in dorsiflexion and plantarflexion were measured in triplicate with the foot placed in 20 degrees of plantar flexion, and the highest value was recorded (24, 25). Peak muscle torque was adjusted for patient age.

#### Cardiopulmonary exercise test

Patients exercised to their maximum ability on an electronically braked cycle ergometer (Ergometrics 800, Sensor-Medics, Yorba Linda, CA). A 12-lead electrocardiogram was obtained at rest in supine, sitting, and standing positions. Three minutes of pedaling in an unloaded state were followed by a ramp increase in work rate to achieve predicted peak work rate in 10–12 min of cycling time (26). Cardiac rhythm and pulse oximetry were monitored throughout the study. Blood pressure was measured at rest and every 3 min during exercise and recovery by auscultation. Metabolic data were obtained throughout the study and for the first 2 min of recovery on a breath-by-breath basis using a metabolic cart (SensorMedics V29, Yorba Linda, CA or similar). Ventilatory anaerobic threshold (VAT) was measured by the V-slope method (27). Peak oxygen consumption (VO_2_) and VO_2_ at VAT were normalized to the percentage expected for age, gender, and body size (28). O_2_ pulse was calculated by dividing peak VO_2_ by maximum heart rate. And expressed in milliliters per beat. A maximal test was defined as a respiratory exchange ratio ≥ 1.10 (29).

#### Exercise cardiac magnetic resonance

Participants ≥ 11 years of age could consent to a non-sedate, resting and exercise CMR protocol (30). The resting protocol consisted of a contiguous axial stack of static steady-state free precession images used for multiplanar anatomic reconstruction, both segmented and free-breathing real time cine short axis stacks, and through-plane retrospectively gated, respiratory-averaged phase-contrast MR across the superior and inferior vena cavae, branch pulmonary arteries, aortic valve, and descending aorta at the level of the diaphragm. After resting CMR image acquisition, the participant was slid partially out from the MR bore to perform lower limb exercise using an MR-compatible supine bicycle ergometer (Lode BV, Groningen, Netherlands). Heart rate was monitored continuously. An initial workload of 20 Watts was increased 20 Watts/minute to achieve the heart rate associated with VAT on the prior CPET. Exercise was suspended, the participant’s feet were removed from the ergometer pedals, and the participant was returned to isocenter for imaging (generally within 5–10 s). A free-breathing real time cine short axis stack was performed; this method has been previously validated against breath-held segmented short axis imaging (31, 32). As breath-holding is not uniform after exercise, respiratory-averaged segmented phase-contrast MR measurements of the aorta, superior vena cava, and descending aorta were performed. Descending aorta flow is substituted for inferior vena cava flow due to difficulty in maintaining inferior vena cava position at exercise (33). Flows and volumes were segmented using cvi42 software 5.13.7 (Circle Cardiovascular Imaging Inc.). Cardiac index was calculated as the product of stroke volume and heart rate, indexed to body surface area. Indexed systemic blood flow was calculated as the sum of superior vena cava and descending aorta flow, indexed to body surface area.

### Statistical analysis

Growth and body composition variables were converted to Z-scores (standard deviation scores) as previously described (7, 21, 34). The 2,000 Centers for Disease Control and Prevention growth charts were used to calculate sex-specific Z-scores for height, weight, and body mass index relative to age (35). Data from > 2,000 healthy, typically developing children from multiple ethnic groups, ages 5–19 years, enrolled in the Bone Mineral Density in Childhood Study (BMDCS) (36, 37), a multicenter longitudinal DXA study, were used to compare participants’ growth Z-scores to a contemporary cohort. These reference data were also used to calculate sex- and race-specific LLMZ relative to age using the LMS method (38). Body composition measures are highly correlated with height and PH physiology is associated with impaired linear growth (39). Therefore, LLMZ was further adjusted for leg length Z-score (40).

Sex-specific reference curves for dominant and non-dominant handgrip were generated using data from the 2011–2012 and 2013–2014 releases of the US National Health and Nutrition Examination Survey (NHANES) using the LMS method (38) and implemented in R programming language using the Generalized Additive Models for Location, Scale, and Shape (GAMLSS library) in R (41).

Standard descriptive statistics [mean ± standard deviation or median (interquartile range)] were used to summarize baseline PH characteristics, LLMZ, muscle strength, quality of life scores, and CPET and eCMR data. Differences in body composition Z-scores between PH participants and the BMDCS reference data were assessed using one-sample Student’s *t*-test. Analyses within the PH group included correlations between LLMZ and continuous variables (e.g., indexed pulmonary vascular resistance) assessed by Pearson or Spearman correlation and comparisons of LLMZ according to categorical variables (e.g., diagnostic group or functional class). Associations between muscle mass and strength, physical activity, and exercise parameters were assessed by Pearson correlation and multiple linear regression. All analyses were conducted using Stata 16.1 with two-sided tests of hypotheses and a *p*-value < 0.05 as the criterion for clinical significance.

## Results

Demographic and clinical characteristics of the 25 participants are displayed in Table 1. The cohort was 56% female and majority white. Mean height Z-score was –0.24 ± 1.24 while mean BMI Z-score was 0.19 ± 0.93. Most patients (64%) were classified as WSPH Group 1 (pulmonary arterial hypertension), but 24% of patients were classified as WSPH group 3 (PH due to lung disease). Of the 16 participants in WSPH Group 1, there were 10 with idiopathic or heritable PAH and 6 with PAH after repair of congenital heart disease. No participants with congenital heart disease had unrepaired cyanotic lesions or significant residual shunts. More patients were WHO functional class I but patients in functional class II enrolled as well. The most common medications were tadalafil and ambrisentan. Twenty-eight percent of the cohort met criteria for vitamin D deficiency. Mean brain natriuretic peptide and hemoglobin levels were within normal limits for our laboratory. Mean tricuspid annular plane systolic excursion Z-score was –2.5 ± 3.2 reflecting decreased right ventricular function. Participants achieved an average 6 MWD of 636 ± 113 meters reflecting good functional status. Cardiac catheterization data were available in 23 participants (90%) at median 1.42 years prior to the study visit. Mean pulmonary artery pressure was 33.1 ± 12.0 mm Hg and indexed pulmonary vascular resistance was 6.2 ± 3.0 indexed Wood units. PAQ scores were lower than reported for healthy populations. Average quality of life score was less than 70 for both participants and parents/guardians.

**TABLE 1:** Demographic and clinical characteristics of participants (*N* = 25).

Variable	N (%) or mean ± SD/median (IQR)
Age, *y*	13.7 ± 2.8
Female	14 (56)
Race	
White	16 (64)
Black/African American	7 (28)
Unknown/not reported	2 (8)
Hispanic or Latino	2 (8)
Height Z-score	–0.24 ± 1.24
Weight Z-score	0.01 ± 1.20
BMI Z-score	0.19 ± 0.93
WSPH classification	
Group 1—PAH	16 (64)
Group 2—PH due to left heart disease	2 (8)
Group 3—PH due to lung disease	6 (24)
Group 4—Chronic thromboembolic PH	1 (4)
WHO functional class	
I	15 (60)
II	10 (40)
Medications	
Sildenafil	2 (8)
Tadalafil	16 (64)
Ambrisentan	15 (60)
Macitentan	1 (4)
Treprostinil SQ	4 (16)
Treprostinil oral	6 (24)
Vitamin OH-D level, *ng/mL*	26.8 ± 13.3
Vitamin D deficiency	7 (28)
BNP, *pg/mL*	22.5 (10.3, 40.1) (Range 10–150.5)
Hemoglobin, *g/dL*	12.9 ± 1.0
TAPSE by echocardiogram, *cm*	1.8 ± 0.5
TAPSE Z-score	–2.5 ± 3.2
6 MWD, *m*	636 ± 113
Cardiac catheterization data	
Interval from study visit, *y*	1.42 (0.54, 1.92)
Mean PA pressure, *mm Hg*	33.1 ± 12.0
PA/Ao ratio	0.5 ± 0.2
Cardiac index, *L/min/m^2^*	3.7 ± 0.8
PVRi, *iWU*	6.2 ± 3.0
PVRi/SVRi ratio	0.3 ± 0.2
Self-reported scores	
PAQ score	1.9 ± 0.6
PCQLI participant score	67.6 ± 17.7
PCQLI parent/guardian score	68.3 ± 16.3

SD, standard deviation; IQR, interquartile range; y, year; BMI, body mass index; WSPH, World Symposium of Pulmonary Hypertension; PAH, pulmonary arterial hypertension; PH, pulmonary hypertension; WHO, World Health Organization; SQ, subcutaneous; BNP, brain natriuretic peptide; TAPSE, tricuspid plane systolic excursion; 6 MWD, 6-min walk distance; PA, pulmonary artery; Ao, aorta; PVRi, indexed pulmonary vascular resistance; SVRi, indexed systemic vascular resistance; PAQ, physical activity questionnaire; PCQLI, pediatric cardiac quality of life inventory.

Skeletal muscle mass and strength data are shown in Table 2. Mean LLMZ was markedly decreased at –0.96 ± 1.14, equivalent to the 17th percentile. Dominant (D-HGZ) and non-dominant (ND-HGZ) were also very low with mean D-HGZ of –1.33 ± 1.37 equivalent to the 9th percentile.

**TABLE 2:** Skeletal muscle assessment.

Variable	Mean ± SD
LLMZ	–0.96 ± 1.14
Hand grip	
D-HGZ	–1.33 ± 1.37
ND-HGZ	–1.14 ± 1.50
Lower extremity biodex, *ft-lbs*	
Knee flexion	25.5 ± 12.3
Knee extension	53.8 ± 23.4
Ankle flexion	15.2 ± 5.4
Ankle extension	29.0 ± 12.1

LLMZ, leg lean mass Z-score; D-HGZ, dominant handgrip Z-score; ND-HGZ, non-dominant handgrip Z-score.

Table 3 includes CPET and eCMR data. Baseline oxygen saturation was 97.7 ± 1.5% with decrease to 91.5 ± 8.3% (range 71–100) with CPET. On CPET, participants performed lower than predicted compared to healthy reference data. Percent predicted peak VO_2_ was approximately 71% while percent predicted VO_2_ at VAT was approximately 80%. Participants only achieved a peak work rate 59% predicted for demographics and body size. Reference data are not available for work rate at VAT. Resting and exercise CMR data are displayed. Right and left ventricular cardiac index and systemic blood flow increased with exercise.

**TABLE 3:** Exercise performance data.

CPET			Mean ± SD
Peak VO_2,_ *mL/kg/min*			29.3 ± 6.6
Percent predicted peak VO_2_			0.71 ± 0.21
O_2_ pulse, *mL/beat*			8.3 ± 2.3
VO_2_ at VAT, *mL/kg/min*			19.3 ± 4.3
Percent predicted VO_2_ at VAT			0.80 ± 0.20
Work, *watts*			92.8 ± 28.3
Percent predicted work			0.59 ± 0.21
Work at VAT, *watts*			44.0 ± 17.2
VE/VCO_2_ ratio			32.6 ± 4.8

**CMR**	**Rest**	**Exercise**	***p*-value for change from rest to exercise**

RV EDVi, *mL/m*^2^	97.9 ± 17.2	88.5 ± 17.8	0.21
RV ESVi, *mL/m*^2^	41.5 ± 9.6	33.4 ± 10.6	0.07
RV SVi, *mL/m*^2^	56.6 ± 13.1	55.0 ± 11.9	0.76
RV EF, %	60.1 ± 10.1	62.6 ± 8.4	0.52
RV cardiac index, *L/min/m*^2^	4.4 ± 1.0	5.7 ± 1.4	0.02
LV EDVi, *mL/m*^2^	81.7 ± 18.9	77.1 ± 20.1	0.57
LV ESVi, *mL/m*^2^	27.8 ± 7.2	20.5 ± 10.0	0.06
LV SVi, *mL/m*^2^	54.0 ± 12.9	56.6 ± 13.1	0.62
LV EF, %	66.1 ± 4.2	73.9 ± 7.6	<0.01
LV cardiac index, *L/min/m*^2^	4.2 ± 0.8	5.9 ± 1.6	<0.01
Systemic flow, *mL/min/m*^2^	4.0 ± 0.8	6.6 ± 1.9	<0.001

CPET, cardiopulmonary exercise test; VO_2_, oxygen consumption; VAT, ventilatory anaerobic threshold; CMR, cardiac magnetic resonance; RV, right ventricular; EDVi, end-diastolic volume indexed; ESVi, end-systolic volume indexed; SVi, stroke volume indexed; EF, ejection fraction; LV, left ventricular.

LLMZ was associated with disease-specific factors. LLMZ was positively associated with hemoglobin (*r* = 0.44, *p* = 0.03) and negatively associated with brain type natriuretic peptide (*r* = –0.40, *p* = 0.05), mean pulmonary artery pressure (*r* = –0.54, *p* < 0.01), and indexed pulmonary vascular resistance (*r* = –0.50, *p* = 0.02) but was not associated with tricuspid annular plane systolic Z-score. LLMZ was higher in those with higher PAQ scores (*r* = 0.50, *p* = 0.01).

**FIGURE 1:**
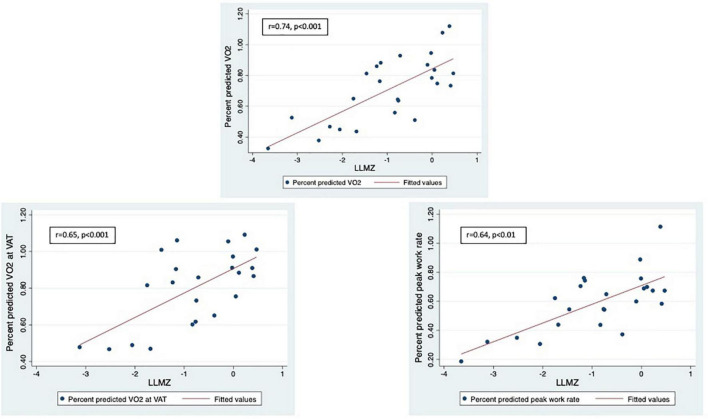
LLMZ, D-HGZ, and PAQ score were associated with percent predicted peak VO_2_, percent predicted VO_2_ at VAT, and percent predicted work. This figure demonstrates the associations between LLMZ and CPET parameters.

**FIGURE 2:**
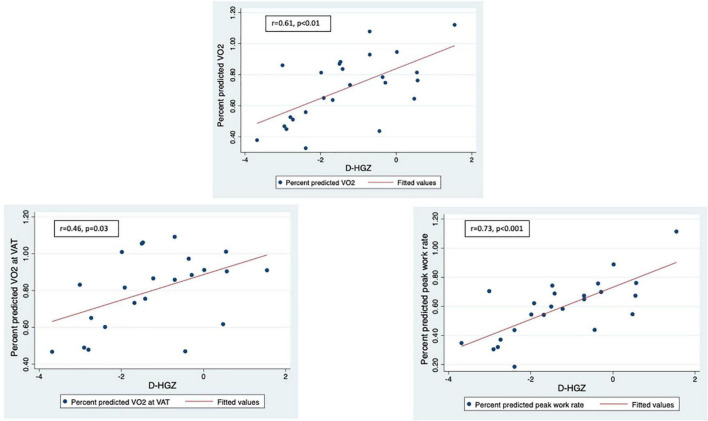
LLMZ, D-HGZ, and PAQ score were associated with percent predicted peak VO_2_, percent predicted VO_2_ at VAT, and percent predicted work. This figure demonstrates the associations between D-HGZ and CPET parameters.

**FIGURE 3:**
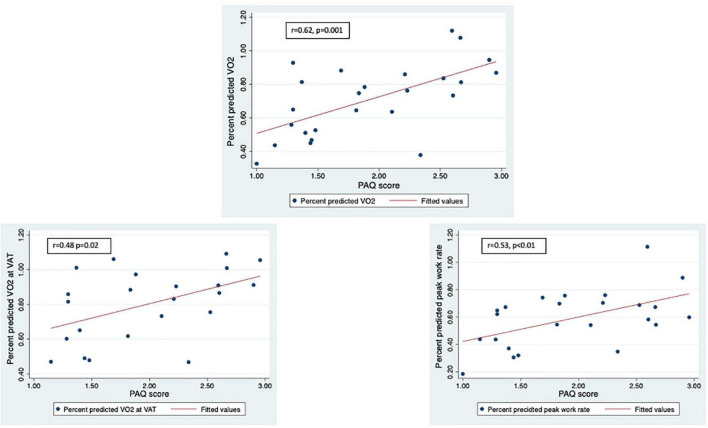
LLMZ, D-HGZ, and PAQ score were associated with percent predicted peak VO_2_, percent predicted VO_2_ at VAT, and percent predicted work. This figure demonstrates the associations between PAQ score and CPET parameters.

LLMZ, muscle strength, and PAQ score were associated with performance on CPET and eCMR (Table 4). LLMZ, both D-HGZ and ND-HGZ, and PAQ score were associated with percent predicted peak VO_2_, percent predicted VO_2_ at VAT, and percent predicted work (Figures 1–3). LLMZ was associated with O_2_ pulse on CPET (*r* = 0.41, *p* = 0.04) while D-HGZ was not. D-HGZ was associated with most recent 6 MWD (*r* = 0.44, *p* = 0.03). D-HGZ, ND-HGZ, and PAQ score were also associated with change in systemic flow from rest to exercise on eCMR, while LLMZ was not (*r* = 0.51, *p* = 0.1).

**TABLE 4:** Associations between skeletal muscle, physical activity, and exercise performance.

Variable	CPET or eCMR parameter	r	*P*-value
**LLMZ**	Percent predicted VO_2_	0.74	<0.001
	Percent predicted VO_2_ at VAT	0.65	<0.001
	Percent predicted work	0.64	<0.01
	O_2_ pulse	0.41	0.04
**D-HGZ**	Percent predicted VO_2_	0.61	<0.01
	Percent predicted VO_2_ at VAT	0.46	0.03
	Percent predicted work	0.73	<0.001
	Change in systemic flow from rest to exercise	0.74	0.02
**ND-HGZ**	Percent predicted VO_2_	0.63	<0.001
	Percent predicted VO_2_ at VAT	0.52	0.01
	Percent predicted work	0.74	<0.001
	Change in systemic flow from rest to exercise	0.70	0.02
**PAQ**	Percent predicted VO_2_	0.62	0.001
	Percent predicted VO_2_ at VAT	0.48	0.02
	Percent predicted work	0.53	<0.01
	Change in systemic flow from rest to exercise	0.70	0.02

CPET, cardiopulmonary exercise test; eCMR, exercise cardiac magnetic resonance; LLMZ, leg lean mass Z-score; VO_2_, oxygen consumption; VAT, ventilatory anaerobic threshold; D-HGZ, dominant handgrip Z-score; ND-HGZ, non-dominant handgrip Z-score; PAQ, physical activity questionnaire.

The associations between lower extremity strength by Biodex and exercise performance were variable. Ankle extension was associated with change in systemic flow from rest to exercise (*r* = 0.69, *p* = 0.04). Both knee extension and flexion were associated with peak work (not percent predicted) (*r* = 0.52, *p* = 0.02 for extension; *r* = 0.48, *p* = 0.03 for flexion), possibly because Z-scores are not available for the Biodex variables.

Results from multiple linear regression models testing the effect of skeletal muscle and physical activity variables on exercise performance are shown in Table 5. LLMZ and PAQ score were positively associated with peak VO_2_, while handgrip Z-score and PAQ score were positively associated with peak work rate. No factors were associated with the change in systemic flow from rest to exercise on eCMR.

**TABLE 5:** Results from multiple linear regression models testing effect of skeletal muscle and physical activity variables on exercise performance.

Variable	Coefficient	Standard error	95% CI	*P*-value
**A. Percent predicted VO_2._**				
LLMZ	0.07	0.03	0.00, 0.14	0.05
PAQ	0.13	0.05	0.02, 0.23	0.02
D-HGZ	0.05	0.02	–0.01, 0.10	0.09
**B. Percent predicted VO_2_ at VAT**				
LLMZ	0.09	0.5	0.0, 0.19	0.06
PAQ	0.10	0.06	–0.03, 0.23	0.12
D-HGZ	0.02	0.03	–0.04, 0.09	0.52
**C. Percent predicted work**				
LLMZ	0.05	0.03	–0.02, 0.11	0.16
PAQ	0.10	0.05	0.01, 0.20	0.04
D-HGZ	0.08	0.02	0.03, 0.13	0.003

(A) Model for percent predicted VO_2_: *R*^2^ = 0.63, *p* < 0.001. (B) Model for percent predicted VO_2_ at VAT: *R*^2^ = 0.42, *p* < 0.01. (C) Model for percent predicted work: *R*^2^ = 0.67, *p* < 0.001.

There were no differences in LLMZ, PAQ, CPET, or eCMR findings in participants with Group 2 PH vs. other classifications. The study findings were unchanged when participants with Group 2 PH were excluded. There were some differences in LLMZ and exercise performance based on medication regimen. Participants on subcutaneous treprostinil had lower LLMZ (–2.02 ± 1.03 vs. –0.76 ± 1.06, *p* = 0.04), percent predicted VO_2_ (0.49 ± 0.11 vs. 0.75 ± 0.21, *p* = 0.02), percent predicted VO_2_ at VAT (0.54 ± 0.13 vs. 0.86 ± 0.17, *p* < 0.01), and percent predicted work (0.41 ± 0.10 vs. 0.63 ± 0.20, *p* = 0.05) compared to those not on subcutaneous treprostinil. Interestingly, participants on oral treprostinil demonstrated greater change in systemic flow with exercise [4.56 ± 1.56 (*n* = 3 with cCMR) vs. 2.02 ± 0.85 (*n* = 7 with eCMR), *p* < 0.01] and higher PAQ (2.3 ± 0.6 vs. 1.8 ± 0.6, *p* = 0.06), D-HGZ (–0.30 ± 1.62 vs. –1.67 ± 1.14, *p* = 0.03), and ND-HGZ (–0.02 ± 1.74 vs. –1.45 ± 1.28, *p* = 0.04) compared to those participants not on oral treprostinil. Participants on ambrisentan demonstrated lower percent predicted VO2 at VAT (0.73 ± 0.22 vs. 0.91 ± 0.13, *p* = 0.04) compared to those not on ambrisentan.

No adverse events occurred with exercise testing.

## Discussion

In this study, we demonstrated marked deficits in skeletal muscle mass and strength in association with worse exercise performance on CPET and eCMR in youth with PH. Lower skeletal muscle mass was associated with physical inactivity. In regression models, skeletal mass, strength, and physical activity were positively associated with exercise performance on CPET. This study builds on our prior work in which lower LLMZ was associated with lower 6 MWD in youth with PH (7). These pilot data add to our appreciation of musculoskeletal abnormalities in pediatric PH and the potential impact of these deficits on exercise performance. Increased understanding of the peripheral determinants of exercise performance may identify targets for intervention trials.

The general “myopathy” seen in adult PH patients is an area of active investigation, but few studies have examined this issue in pediatric PH. PH is increasingly understood to be a systemic condition with metabolic, inflammatory, genetic, and epigenetic contributions (42). As one of the extra-cardiopulmonary manifestations of this condition, skeletal muscle dysfunction can be grouped into structural deficits, functional impairment, and molecular abnormalities with evidence from both human and animal studies (42). Structural deficits include reduced muscle fiber cross sectional area, lower proportion of type I fibers, decreased capillary density, lipid inclusion, and loss of mitochondrial structure (8, 10, 43). Functional impairment can manifest as reduced muscle strength, decreased muscular endurance, impaired oxygenation in the microcirculation, reduced type I fiber tension, and sarcomeric dysfunction (6, 8, 9, 44, 45). Molecular abnormalities have been described including reduced oxidative capacity, increased protein degradation and decreased protein synthesis, impaired angiogenesis, and impaired mitochondrial function (11, 46, 47). The spectrum of muscular abnormalities in children with PH is unknown. As pediatric PH often occurs in the context of congenital heart disease, developmental lung diseases, genetic differences, and other syndromic conditions, the findings may be even more severe. To our knowledge, no other investigators have explored decreased muscle mass and strength in children with PH. The data are essential to understanding the burden of disease across the lifespan and identifying appropriate timepoints for intervention. Future studies should explore the breath of these findings.

We demonstrated associations between muscle deficits and performance on CPET. CPET is a comprehensive assessment of a patient’s exercise performance that utilizes inspiratory and expiratory gas exchange to quantify peak oxygen consumption, carbon dioxide production, and minute ventilation (48). Peak VO_2_ is the most common indicator of a patient’s cardiorespiratory fitness. Adult patients with PH demonstrate decreased peak VO_2_, higher VE/VCO_2_ (ventilatory equivalents of carbon dioxide) indicating ventilatory inefficiency, lower arterial CO_2_ tension and end-tidal CO_2_ tension, lower O_2_ pulse (VO_2_/heart rate, a surrogate for stroke volume), and lower systemic oxygen saturation (49–51). Worse peak VO_2_ is associated with more symptoms of dyspnea and fatigue which limit quality of life. Both low peak VO_2_ and low 6 MWD are associated with mortality in adults with PAH and are incorporated into REVEAL 2.0 and European Society of Cardiology/European Respiratory Society risk stratification tools (52, 53). However, completion of a CPET requires significant developmental skills and reference values relative to outcomes are lacking in pediatric PH (54, 55). The lack of adverse events in this pilot study suggests that select older children and teenagers with PH can safely perform CPET and eCMR. Peak VO_2_ is included in pediatric PAH disease severity guidelines (56) and perhaps exercise parameters will be incorporated into novel pediatric risk stratification tools which are being developed. To our knowledge, this is the first study to express PH participants’ CPET performance relative to reference values from more than 1,800 healthy United States children published by members of our study team (28). Participants in our study generally performed at 60–80% predicted, suggesting children with PH already face marked limitations prior to progressive decline in adulthood. Additionally, this is the first report of eCMR in pediatric PH, although we have previously described eCMR in patients with single ventricle physiology after Fontan palliation (21). Higher PAQ and handgrip Z-scores were associated with greater change in systemic flow from rest to exercise, raising compelling questions regarding physical activity, strength, and the ability to augment systemic flow on exertion. Future, larger studies should also explore the correlation between eCMR performance and PH disease-specific outcomes.

While peak VO_2_ is a common outcome reported in research and clinical care, CPET measures of submaximal performance including VO_2_ at VAT and peak work rate relative to respiratory exchange ratio as well as measures of anaerobic strength may better reflect a patient’s daily activities and may also be targets for improvement. PH patients rapidly reach anaerobic threshold when performing activities of daily living such as doing laundry and folding clothes, cooking and setting the table, and walking outside with moderate effort (57, 58). These activities are often performed with short bursts of activity. The associations between muscle deficits, VO_2_ at VAT, and peak work rate in our study suggest that skeletal muscle deficits may impact everyday activity performance for PH patients and improvements in muscle mass or function could improve quality of life and other outcomes over longer periods. These findings are also consistent with our previous report associating lower LLMZ with lower 6 MWD, another test of submaximal exercise performance (7).

Cardiopulmonary rehabilitation improves symptoms and functional status in adult PH but the mechanisms underlying those improvements are incompletely understood. Some studies have demonstrated improvement in functional status and exercise performance without change in pulmonary hemodynamics. Data such as those from Bauer et al. (6) and Mainguy et al. (10) have supported development of exercise rehabilitation programs to improve muscle function and strength. In a separate study, Mainguy demonstrated increased 6 MWD, increased exercise duration on CPET, and improved VE/CO_2_ in association with decreased number of quadriceps type IIx fibers in 5 adult IPAH patients after a 12-week rehabilitation program (59). De Man also demonstrated increased quadriceps strength and endurance, increased quadriceps capillarization, and higher oxidative enzyme activity in 19 adult IPAH patients after a 12-week rehabilitation program (60). There is only one published report of exercise training in pediatric PH. Zöller et al. demonstrated increased treadmill running distance and improved VO_2_ at VAT after a 16-week home exercise program in 9 children with PH and functional class I and II (61). Skeletal muscle characterization was not part of that study. While PH patients perform less moderate to vigorous physical activity compared to healthy peers (62), no studies have tested interventions to improve activity. To our knowledge, no pediatric PH exercise trials have described changes in physical activity and/or skeletal muscle performance with training. Future studies should determine whether low skeletal muscle mass is a marker of disease severity or a modifiable determinant of exercise performance in youth with PH. It is not known whether increasing physical activity can augment skeletal muscle characteristics and improve exercise performance in youth with PH.

A primary limitation of this study is the lack of control group. It is not clear whether the relationships between LLMZ, PAQ score, and exercise performance are different in PH patients vs. healthy youth. Additionally, causal inferences cannot be made from these data given the study’s cross-sectional design. The small number of participants with certain medication regimens does not allow us to draw conclusions regarding relationships between medications, underlying disease status, and the study outcomes. The relationship between disease progression, muscle deficits, and exercise performance is also unknown. The study was also limited by lack of comparison reference data for lower extremity strength. The lack of association between leg strength and exercise performance may have been because we were unable to express strength relative to gender and body size. Finally, physical activity was measured by self-report. The analyses would have been strengthened by quantification of physical activity by wearable accelerometer. However, despite these limitations, this study has generated preliminary data for future observational studies and interventional trials.

## Conclusion

Youth with PH demonstrate inactivity and marked skeletal muscle deficits in association with worse exercise performance on CPET and eCMR. Future studies should determine whether low skeletal muscle mass is a marker of disease severity or a modifiable target for exercise interventions. Interventions that improve skeletal muscle mass and function could improve exercise performance in this population.

## Data availability statement

The raw data supporting the conclusions of this article will be made available by the authors, without undue reservation.

## Ethics statement

The studies involving human participants were reviewed and approved by the Children’s Hospital of Philadelphia Institutional Review Board. Written informed consent to participate in this study was provided by the participants or their legal guardian/next of kin.

## Author contributions

CA designed the study, recruited participants, supervised study procedures, analyzed and interpreted data, and wrote the manuscript. MM and SP supervised exercise testing procedures, interpreted exercise data, and reviewed the final manuscript. MH, KW, and MF supervised exercise cardiac magnetic resonance procedures, analyzed and interpreted the cardiac magnetic resonance data, and reviewed the final manuscript. BZ analyzed and interpreted anthropometry and densitometry data and reviewed the final manuscript. All authors contributed to the article and approved the submitted version.

## Abbreviations

6 MWD: 6-min walk distance
BMDCS: Bone Mineral Density in Childhood Study
CPET: cardiopulmonary exercise test
DXA: densitometry
eCMR: exercise cardiac magnetic resonance
IPAH: idiopathic pulmonary arterial hypertension
LLMZ: leg lean mass Z-score
NHANES: National Health and Nutrition Examination Survey
PAQ: physical activity questionnaire
PH: pulmonary hypertension
VAT: ventilatory anaerobic threshold
VE/VCO_2_: ventilatory equivalents of carbon dioxide
VO_2_: oxygen consumption.
